# Comparison of second molar protraction using different timing for piezocision application: A randomized clinical trial

**DOI:** 10.1590/2177-6709.27.4.e2220503.oar

**Published:** 2022-09-23

**Authors:** Elham S. ABU ALHAIJA, Marwan M. AL-AREQI, Emad F. AL MAAITAH

**Affiliations:** 1 Qatar University, QU Health, College of Dental Medicine (Doha, Qatar).; 2 Jordan University of Science and Technology, Faculty of Dentistry, Department of Preventive Dentistry (Irbid, Jordan).

**Keywords:** Piezocision, Molar protraction, Timing

## Abstract

**Objective:**

To compare second molar protraction between early, late and no piezocision groups.

**Material and Methods:**

Forty subjects with bilaterally extracted mandibular first molars were selected to participate in the study. Subjects were subdivided into two groups: piezocision and no piezocision. The piezocision group was further subdivided into two subgroups: early piezocision (piezocision performed immediately before second molar protraction) and late piezocision (piezocision performed three months after starting molar protraction). In the no piezocision group, molar protraction was done without surgery. The intervention (piezocision group and timing of piezocision/side within group) was randomly allocated using the permuted random block size of 2, with 1:1 allocation ratio. The amount of second molar protraction, duration of space closure and anterior anchorage loss were measured. A repeated measures analysis of variance was conducted to define the differences between the measured variables at the different time intervals. Differences between groups were assessed using ANOVA test.

**Results:**

No difference was detected between early and late piezocision groups in the amount of molar protraction at the end of space closure. Duration of complete space closure was 9 and 10 months in the piezocision and no piezocision groups. Anchorage loss was similar between the three studied groups.

**Conclusions:**

Early and late piezocision have similar effect and both increased the amount of second molar protraction temporarily in the first 2-3 months after surgery. Duration of mandibular first molar space closure was reduced by one month when piezocision was applied. Anchorage loss was similar in the three groups.

## RESUMO

**Objetivo:**

Comparar o efeito dos tempos de aplicação precoce, tardia e sem piezocisão, na protração de segundos molares.

**Métodos:**

Quarenta indivíduos com os primeiros molares inferiores extraídos bilateralmente foram selecionados para participar do estudo, sendo divididos em dois grupos: piezocisão e sem piezocisão. O grupo de piezocisão foi subdividido em dois subgrupos: piezocisão precoce (piezocisão realizada imediatamente antes da protração de segundos molares) e piezocisão tardia (realizada três meses após o início da protração dos molares). No grupo sem piezocisão, a protração de molares foi feita sem cirurgia. A intervenção (grupo de piezocisão e momento da piezocisão/lado dentro do grupo) foi alocada aleatoriamente usando o tamanho de bloco aleatório permutado de 2 com proporção de alocação de 1:1. A quantidade de protração de segundos molares, tempo para fechamento de espaços e perda de ancoragem anterior foram medidos. Uma análise de variância para medidas repetidas foi realizada para definir as diferenças entre as variáveis medidas nos diferentes intervalos de tempo. As diferenças entre os grupos foram avaliadas pelo teste ANOVA.

**Resultados:**

Não foi detectada diferença entre os grupos de piezocisão precoce e tardia, em relação à quantidade de protração de molares ao fim do fechamento dos espaços. O tempo para o fechamento completo do espaço foi de nove e dez meses nos grupos piezocisão e sem piezocisão, respectivamente. A perda de ancoragem foi semelhante nos três grupos avaliados.

**Conclusões:**

As piezocisões precoce e tardia têm efeito semelhante e ambas aumentaram temporariamente a quantidade de protração dos segundos molares nos primeiros dois a três meses após a cirurgia. A duração do fechamento de espaço dos primeiros molares inferiores foi reduzida em um mês quando a piezocisão foi aplicada. A perda de ancoragem foi semelhante nos três grupos.

## INTRODUCTION

Fixed appliance orthodontic treatment of moderate to severe malocclusions lasts a mean duration of 18 months.^1^ In a systematic review, Mavreas and Athanasiou^2^ concluded that the duration of orthodontic treatment is affected by several factors: extraction of teeth, type and severity of malocclusion, timing of treatment and the compliance of the patients. Recently, the acceleration of orthodontic treatment started to gain interest by both patients and orthodontists, especially adult patients, who prefer to complete orthodontic treatment as fast as possible. Also, a shorter treatment duration has the advantage of reducing many side effects associated with fixed orthodontic treatment, such as dental caries, gingival recession and root resorption.^3^ ^,^ ^4^

Piezocision to accelerate tooth movement is a localized piezoelectric alveolar decortication technique that combines buccal microincisions and minimally invasive corticotomies.^5^ Although many clinical trials were conducted to evaluate the effect of piezocision on the rate of tooth movement,^6^ ^-^ ^15^ there is still a controversy regarding the acceleratory effect of piezocision on orthodontic tooth movement. Some studies reported a decrease in treatment duration, ranging from 23% to 59%, in the piezocision groups.^8^ ^,^ ^11^ ^-^ ^13^ On the other hand, Tunçer et al.^10^ reported similar rate and treatment duration between piezocision and control groups. Al-Areqi et al.^15^ found that overall second molar protraction was accelerated by only one month. Differences in the sample size, piezocision surgical design and location, the tooth to be moved, stage of orthodontic treatment and mechanics used in the aforementioned studies contributed to this inconsistency.

The extraction space of mandibular first molar is considered relatively large. This may affect the archwire sliding during space closure and results in archwire binding, making space closure a more time-consuming procedure. Therefore, the timing for performing piezocision, whether it is before starting space closure or after closing part of the space, may produce different acceleratory effect on mandibular second molar protraction. This has not been investigated so far in orthodontic literature; therefore, the purpose of this randomized clinical trial is to investigate and compare the amount of mandibular second molars protraction when piezocision is performed earlier (immediately before molar protraction, larger extraction space) or later (after three months of molar protraction, shorter extraction space), to find out the proper timing for piezocision procedure. The null hypothesis of no difference in the amount of second molar protraction among early piezocision, late piezocision and control (no piezocision) groups was tested. 

## MATERIAL AND METHODS

### TRIAL DESIGN

This study was a randomized controlled clinical trial with a 1:1 allocation ratio (piezocision *vs.* no-piezocision). In the piezocision group, a split-mouth design was utilized, with the timing for piezocision (early or late) randomly assigned to the left or right sides of treated patients. The methods were not changed after trial initiation.

### PARTICIPANTS, ELIGIBILITY CRITERIA AND SETTINGS

The study was reviewed and approved by the Institutional Review Board (approval number 28/98/2016) at the University Hospital of Jordan University of Science and Technology (JUST). This trial was registered at ClinicalTrials.gov with identifier number NCT04338789. The participants for this study were recruited from patients attending orthodontic clinics at the postgraduate dental clinics of JUST. All surgical procedures and orthodontic treatments were undertaken at the postgraduate dental clinics of JUST. The inclusion criteria for this study were: age range from 18 to 30 years, bilaterally extracted mandibular first molars (first molars extracted more than one year ago and with a residual extraction space of more than 5 mm), Class I malocclusion with molar protraction indication, and all permanent teeth present, except for the extracted mandibular first molars. Exclusion criteria included: poor oral hygiene, history of previous orthodontic treatment, any systemic diseases and smoking.

Subjects were selected based on these inclusion criteria, and were asked to sign a consent form to participate in this study, after clarifying the purpose of the intervention. Initial records (orthopantomogram, lateral cephalogram and alginate impressions) were obtained for all participants. Subjects were referred to the Periodontics department for evaluation of their periodontal health and to have regular oral care thereafter. 

### SAMPLE SIZE CALCULATION

Sample size was calculated using the G*Power v. 3.1.9 program. According to the power analysis and assuming a small effect size difference (0.25) between groups, based on a split-mouth study to compare the monthly rate of molar protraction,^15^ the power analysis yielded a total sample size estimate of 39 molars at a conventional alpha level (0.05) and desired power (1 - β) of 0.95. Assuming an overall attrition rate of 15%, initial recruitment should target a total of 45 molars with 15 molars per group. 

### RANDOMIZATION

Random allocation of subjects according to their group (piezocision or no piezocision) was done using the permuted random block size of 2, with a 1:1 allocation ratio, by one dental assistant. The allocation sequence was concealed from the researcher by sequentially numbered, opaque, sealed and stapled envelopes before the intervention. Patients were asked to pick randomly a sealed envelope that assigned the method of intervention. 

In piezocision group, just before mandibular molar protraction, early piezocision was randomly assigned to patients’ left or right side by the same dental assistant, with the contralateral side allocated to serve in the other group (late piezocision). Patients were asked to pick randomly a sealed envelope that assigned the side of intervention (early piezocision). 

### BLINDING

Blinding of either patient or clinician was not possible. However, the measurements of the dental casts were performed by one research assistant who was blinded to the type of the intervention used.

### INTERVENTION

#### Orthodontic intervention

The selected patients had their orthodontic treatment performed by the same orthodontic resident, using fixed preadjusted Edgewise-orthodontic appliances (3M Gemini Unitek brackets; 0.022-in Roth prescription). Patients were monitored with monthly appointments. Tooth alignment started with an 0.014-in nickel-titanium (NiTi) archwire, followed by a sequence of 0.016-in, 0.018-in, 0.016 x 0.022-in and 0.019 x 0.025-in NiTi archwires, before a 0.019 x 0.025-in stainless steel (SS) rectangular archwire was tied into the slot. The patients were divided into three groups: 

#### Group 1: Early piezocision/molar protraction

This group consisted of 20 patients (17 females and 3 males; aged 21.25 ± 2.10years) with left or right mandibular first molar extraction. In this group, piezocision was performed immediately before second molar protraction (inter bracket/tube span 10.21 ± 0.84mm). 

#### Group 2: Late piezocision/molar protraction

This group consisted of 20 patients (17 females and 3 males, aged 21.25 ± 2.10years) with left or right mandibular first molar extraction. In this group, piezocision was performed three months after starting molar protraction (Inter bracket/tube span at time of surgery 7.10 ± 0.79mm). 

#### Group 3: No piezocision /molar protraction (control group)

This group consisted of 20 patients (15 females and 5 males, aged 22.14 ± 1.53 years) with bilateral first molar extraction spaces. In this group, subjects refused to perform piezocision. This group served as a control, and molar protraction was carried out with no piezocision (inter bracket-tube span at time of protraction 10.27 ± 0.73mm).

### MOLAR PROTRACTION

After tying an 0.019 x 0.025-in SS archwire, a miniscrew (3M Unitek™) used as Temporary Anchorage Device (TAD), with a 1.8-mm diameter and 8-mm length, was screwed through the bone on the labial surface of the mandibular alveolar ridge between the roots of mandibular canine and first premolar in all the patients. A NiTi coil spring (3M) was used for space closure (150g) and was attached from the mandibular second molar hook to the head of the miniscrew. Labial movement of mandibular incisors during molar protraction was prevented by lingual incisors crown torque and cinch-back of the archwire. Occlusal interferences were checked regularly and if present, glass ionomer cement was used on the maxillary incisors to raise the bite. Patients were followed-up on a monthly basis, during which, alginate impressions were obtained for all patients at each visit, after removal of the mandibular archwire. Study models were then fabricated. The time points for the protraction rate were; T_0_: baseline measurement before protraction,T_1_: after one month,T_2_: after two months,T_3_: after three months, T_4_: after four months,T_5_: after five months,T_6_: after six months and T_10_: after ten months of molar protraction.

### SURGICAL PROCEDURE - PIEZOCISION

All the piezocisions were performed by a single resident in the periodontal clinic. The patients were asked to rinse with 0.2% chlorhexidine gluconate for one minute before being given local anesthesia. Then, 2% lidocaine anesthetic agent was used to perform an infiltration technique mesial and distal to the mandibular first molar extraction space. After that, two incisions were made using a #15 blade, mesial and distal to the extraction space. A piezotome was then inserted into the previously-made incisions and bone cuts were done up to the mucogingival line, at a depth of 3 mm. Piezocision was performed using a Mectron Piezosurgery device (Mectron, Genova, Italy). No sutures or any surgical dressings were placed after. In Groups 1 and 2, patients were asked to return to the orthodontic clinic, immediately after the piezocision procedure, to attach the NiTi coil spring from the hook of the mandibular permanent second molar to the miniscrew. In Group 3, a NiTi coil spring was attached from the hook of the mandibular permanent second molar to the miniscrew once the 0.019 x 0.025-in SS archwire was tied-in.

## OUTCOMES

### PRIMARY OUTCOMES

#### Second molar protraction

Measured monthly as the distance from a point representing the mesial surface of the mandibular second molar at cementoenamel junction (CEJ) to a point representing the miniscrew head, constructed on the mandibular occlusal plane. It was determined by direct measurement of the study casts. 

#### Anchorage loss

» Mandibular incisors: the change in mandibular incisor inclination post-treatment, from lateral cephalogram (mandibular incisors/mandibular plane).

» Mandibular second premolar: The distance from the distal surface of the mandibular second premolar at CEJ to the miniscrew, as determined from direct measurement of the study casts. 

### SECONDARY OUTCOME

#### Treatment duration

Determined in months from the start of mandibular molar protraction until first molar space was almost or completely closed.

### METHOD ERROR

Ten subjects were randomly selected, and the study models measurements were done twice with two-week interval. The Dahlberg formula was used to calculate the standard error of the method. Dahlberg errors were 0.17mm for the amount of molar protraction, 0.21mm for second premolar distal movement, and 0.37^ο^ for mandibular incisor inclination. 

### INTERIM ANALYSES AND STOPPING GUIDELINES

Not applicable

### STATISTICAL ANALYSIS (PRIMARY AND SECONDARY OUTCOMES, SUBGROUP ANALYSES)

Statistical analysis was performed using the Statistical Package for the Social Sciences computer software (SPSS v. 22.0, SPSS Inc., NY, USA). Descriptive statistics were calculated for all the measured variables for each group. Intention to treat (ITT) analysis was performed. A repeated measures analysis of variance (within-subject’s ANOVA) test was conducted to examine and define the differences between the measured variables at the different time intervals. Differences between groups were assessed using ANOVA. Bonferroni *post-hoc* multiple comparisons test was used to identify differences between the groups. The level of significance was set at *p*≤0.05).

## RESULTS

**Figure 1:**
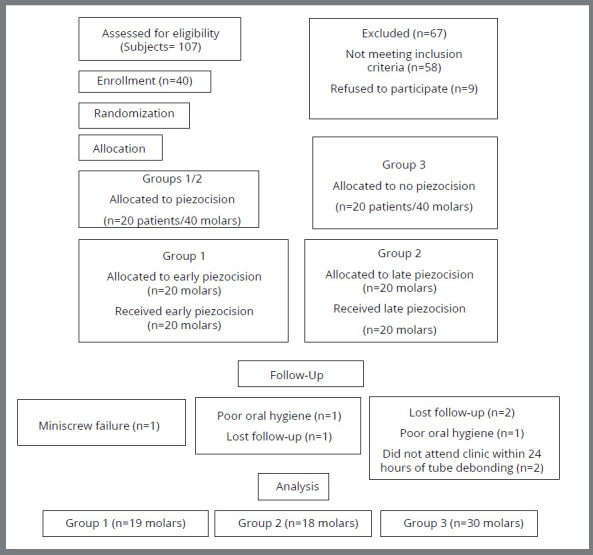
CONSORT flowchart showing patient flow during the trial.

Participant flowchart can be seen in Figure 1. Subjects were recruited between December 2016 and June 2018, with the final data collection in December 2019. In Group 1, 20 patients/molars received early piezocision; one patient was excluded (1 miniscrew failure). In Group 2, 20 patients/molars received late piezocision; two patients were excluded (1 missed appointments and 1 had poor oral hygiene). In Group 3, five patients (10 molars) were excluded (2 missed appointments, 1 had poor oral hygiene, 2 patients did not attend within 24 hours of bracket debonding during protraction). During the analysis stage, there were records for 34 patients (27 females and 7 males) with 67 first molar extraction spaces (19 patients/molars received early piezocision intervention, 18 patients/molars received late piezocision intervention and 15 patients/30 molars did not receive any surgery and acted as the control group). Bracket or molar tube failure during molar protraction was identified in 2 patients only in Group 3, who were excluded from the final analysis. The endpoint of this study was complete mandibular first molar space closure.

## BASELINE DATA

Data regarding age and cephalometric analysis of the subjects in each group are listed in Table 1. 

**Table 1:** Baseline characteristics of subjects included in the final analysis.

	Groups 1 and 2	Group 3
Age (years)	21.25 ± 2.10	22.14 ± 1.53
SNA (degrees)	80.23 (1.56)	79.40 (1.93)
SNB (degrees)	77.50 (1.06)	76.32 (1.40)
ANB (degrees)	3.11 (0.41)	3.06 (0.72)
Maxillary/Mandibular planes angle (degrees)	25.76 (1.63)	24.06 (3.57)
Mandibular incisors/Mandibular plane angle (degrees)	93.67 (7.43)	92.79 (5.69)

### NUMBERS ANALYZED FOR EACH OUTCOME

#### Rate of mandibular molar protraction and anchorage loss

First month after protraction (T_1_ in Groups 1 and 3; T_4_ in Group 2), 1 molar from Group 1, 1 molar from Group 2, and 3 patients from Group 3 were excluded (n = 19 in Group 1, n = 19 in Group 2 and n = 34 in Group 3). On the second and third months after protraction (T_2_ and T_3_ in Groups 1 and 3; T_5_ and T_6_ in Group 2), one patient from Group 2 and 2 patients from Group 3 did not show up for evaluation (n = 19 in Group 1, n = 18 in Group 2 and n = 30 in Group 3). During the analysis stage (T_10_), there was full data for 19 molars in Group 1, 18 molars in Group 2 and 30 molars in Group 3. 

### PRIMARY OUTCOMES

#### Amount of mandibular molar protraction

Means, standard deviations, differences between the means and *p*-values for the rate of second molar protraction and anchorage loss in the studied groups at the different time points are shown in Tables 2, 3 and 4. 

**Table 2:** Means and standard deviations (SD) for the distance from mandibular second molar to the miniscrew at the different time intervals in the studied groups.

Time interval	Early piezocision Group 1 / Mean ± SD	Late piezocision Group 2 / Mean ± SD	No piezocision Group 3 / Mean ± SD
Baseline (T0)	23.11 ± 1.04	23.23 ± 1.01	23.17 ± 0.93
After 1 month (T1)	21.94 ± 1.07	22.53 ± 0.97	22.49 ± 0.94
After 2 months (T2)	20.88 ± 1.18	21.89 ± 0.99	21.88 ± 1.05
After 3 months (T3)	20.10 ± 1.18	21.15 ± 1.00	21.25 ± 1.01
After 4 months (T4)	19.36 ± 1.31	19.62 ± 1.06	20.58 ± 0.99
After 5 months (T5)	18.68 ± 1.18	18.55 ± 1.16	19.98 ± 1.00
After 6 months (T6)	18.09 ± 1.17	17.72 ± 1.24	19.34 ± 0.98
After 10 months (T10)	15.79 ± 1.04	15.59 ± 1.01	16.09 ± 1.17

**Table 3:** Mean differences, standard deviations (SD) and differences between means for the amount of mandibular molar protraction at the different time intervals and duration of space closure in the studied groups.

Molar protraction	Early piezocision Group 1 Diff between means (SD)	Late piezocision Group 2 Diff between means (SD)	No piezocision Group 3 Diff between means (SD)	Groups 1 & 2	Groups 1 & 3	Groups 2 & 3
Diff T1-T0	-1.17 ± 0.20	-0.70 ± 0.27	-0.68 ± 0.19	***	***	NS
Diff T2-T1	-1.06 ± 0.25	-0.64 ± 0.21	-0.61 ± 0.23	***	***	NS
Diff T3-T2	-0.78 ± 0.26	-0.74 ± 0.13	-0.63 ± 0.22	NS	NS	NS
Diff T4-T3	-0.74 ± 0.28	-1.53 ± 0.39	-0.66 ± 0.21	***	NS	***
Diff T5-T4	-0.68 ± 0.22	-1.07 ± 0.27	-0.61 ± 0.24	***	NS	***
Diff T6-T5	-0.59 ± 0.16	-0.83 ± 0.15	-0.64 ± 0.14	***	NS	***
Diff T3-T0	-3.01 ± 0.45	-2.08 ± 0.48	-1.92 ± 0.46	***	***	NS
Diff T6-T3	-2.00 ± 0.52	-3.43 ± 0.64	-1.90 ± 0.39	***	NS	***
Diff T6-T0	-5.02 ± 0.32	-5.52 ± 0.41	-3.83 ± 0.50	***	***	***
Diff T10-T0	-7.33 ± 0.56	-7.65 ± 0.42	-6.28 ± 0.68	NS	***	***
Space closure duration (month)	9.33 ± 0.76	9.26 ± 0.86	10.17 ± 0.89	NS	***	***

NS = not significant, *** p < 0.001.

**Table 4:** Means, standard deviations (SD) and differences between means for anchorage loss variables in the studied groups.

	Early piezocision Group 1 Diff between Means (SD)	Late piezocision Group 2 Diff between Means (SD)	No piezocision Group 3 Diff between Means (SD)	Groups 1 & 2	Groups 1 & 3	Groups 2 & 3
MANDIBULAR SECOND PREMOLAR DISTAL MOVEMENT (mm)						
T0 (Baseline)	14.56 ± 0.58	14.77 ± 0.59	14.73 ± 0.61	NS	NS	NS
T3 (after 3 months)	14.94 ± 0.61	15.03 ± 0.55	15.15 ± 0.67	NS	NS	NS
T6 (after 6 months)	15.24 ± 0.61	15.31 ± 0.61	15.46 ± 0.68	NS	NS	NS
T10 (after 10 months)	15.49 ± 0.70	15.69 ± 0.65	15.73 ± 0.76	NS	NS	NS
Diff T3-T0	0.38 ± 0.25	0.28 ± 0.18	0.41 ± 0.21	NS	NS	NS
Diff T6-T3	0.29 ± 0.18	0.28 ± 0.19	0.31 ± 0.16	NS	NS	NS
Diff T10-T0	0.92 ± 0.34	0.94 ± 0.40	0.99 ± 0.32	NS	NS	NS
	Groups 1 & 2 (piezocision) Means (SD)		Group 3 (no piezocision) Means (SD)		Diff between means (SE)	
MANDIBULAR INCISORS INCLINATION						
Pretreatment	93.67 ± 7.43		92.79 ± 5.69		0.88 (2.47)	
Post-treatment	96.00 ± 9.20		96.57 ± 6.23		0.57 (2.94)	
Diff post- pre-treatment	2.33 ± 3.77		3.79 ± 2.36		1.45 (1.16)	

NS = not significant.

In the early and late piezocision groups, the rate of molar protraction was increased in the first two months after performing piezocision, and slowed down afterwards. Molar protraction three months after early piezocision was 3.01 mm, 2.08 mm and 1.92 mm in the early, the late and the no piezocision groups, respectively. Significant differences were detected between the early piezocision group and the other two groups (*p*>0.001). Three months after late piezocision, molar protraction was 2.00mm, 3.43mm and 1.91mm in the early, the late and the no piezocision groups, respectively. Significant differences were detected between the late piezocision group and the other two groups (*p*>0.001).

Six months after initial space closure, the amount of molar protraction was 5.02mm, 5.52mm and 3.83mm in Groups 1, 2 and 3, respectively. Significant differences were detected between the three studied groups (*p*>0.001). However, near to the end of space closure (10 months), no difference was detected between early and late piezocision groups regarding the amount of molar protraction (*p*>0.05).

### ANCHORAGE LOSS

In piezocision groups, mandibular incisors proclined by 2.33^ο^, whereas in no piezocision group, mandibular incisors proclined by 3.79^ο^ post-treatment. The difference was not statistically significant (*p*>0.05). Mandibular second premolar distal movements were 0.92mm, 0.94mm and 0.99mm in the early, the late and the no piezocision groups, respectively. No significant differences were detected between the three studied groups (*p*>0.05).

### SECONDARY OUTCOME

Duration of mandibular first molar space closure (Table 3) was 9.33 months, 9.26 months and 10.17 months in the early, the late and the no piezocision groups, respectively. Although treatment duration in the no piezocision group was one month more than the piezocision groups, the differences were statistically significant (*p*<0.01). However, duration of first molar space closure was similar in the early and late piezocision groups (*p*>0.05). 

### HARMS

No negative outcomes were reported by any patient during the trial.

## DISCUSSION

In orthodontic literature, there is conflicting evidence regarding the effect of inter-brackets distance on frictional forces during space closure. Some investigators reported that an increased inter-brackets width during space closure allow a greater tipping, which could lead to increased angle interface between the archwire and the bracket floor, resulting in a greater binding incidence,^16^ while others stated that resistance to sliding is inversely proportional to inter-brackets distance.^17^ ^,^ ^18^ During mandibular second molar protraction, first molar extraction space will be large initially and will be reduced during subsequent space closure. To our knowledge, no study investigated the effect of inter-brackets distance on the rate of tooth movement, therefore, the present randomized controlled clinical study was conducted. 

A split-mouth design was adopted in this study to reduce the biological variability between the subjects^19^ in which piezocision was applied, according to the technique described by Dibart et al.^5^ In the current study, 3-mm vertical cuts in the buccal side of alveolar ridge were performed. The cuts were deeper than the traditional circumscribed corticotomy, which involves 2-mm vertical and horizontal cuts in the cortical bone circumscribing the teeth to be moved, to ensure blade access to the cortical bone.^5^

Space closure was carried out on a rigid rectangular SS archwire to achieve maximum amount of bodily movement.^20^ However, mesial tipping of mandibular second molars may still occur due to the play between archwire and molar tube. A closed NiTi coil spring was used to achieve molar protraction, since it provides constant force, when compared to an elastomeric chain,^21^ and provides a more predictable amount of force.

The current trial demonstrated a significant increase in the rate of mandibular molar protraction in patients who received piezocision, compared to no piezocision group, which lasted for 2 to 3 months in the early and late groups, respectively. The increased rate of tooth movement found in this trial was in agreement with previous studies.^6-8,11,13,14^ Alfawal et al.^11^ reported that the piezocision side exhibited a two-fold greater canine retraction rate in the first month and a 1.5-fold in the second month before declining toward the normal value at the end of 3 months. Also, Charavet et al.^14^ found that piezocision was effective during three months after surgery. On the other hand, Tunçer et al.^10^ found no difference in the rate of tooth movement between piezocision and no piezocision groups. Also, in a systematic review, Mheissen et al.^22^ demonstrated that the evidence that piezocision accelerates tooth movement is low and is not clinically significant. 

Up to 1-mm increase in the rate of molar protraction was found in the current study, which was less than that reported in previous trials. This may be related to different factors, such as: the structure of mandibular bone, compared to maxillary bone; molar protraction through old extraction space, compared to retraction of canine to the recently extracted first premolar space. Charavet et al.^14^ reported that piezocision is more effective in the maxilla than in the mandible. 

In the current study, the rate of molar protraction was almost similar whether the inter-brackets distance was small or large. In both early and late piezocision groups, tooth movement was accelerated temporarily for two months by a comparable amount. The findings of the current study was in disagreement with previous studies that suggested inter-brackets width affects frictional resistance. Some studies reported that reduced inter-brackets distance produced greater frictional resistance,^17^ ^,^ ^18^ ^,^ ^23^ and others suggested that as the inter-brackets distance reduces, friction reduces, due to reduction in tipping.^24^ ^,^ ^25^ The findings of the current study may be related to the other variables affecting force delivery during molar protraction. Nanda^26^ stated that a large inter-brackets distance reduces the load/deflection rate and helps deliver constant force magnitude, providing better directional control of the tooth movement. 

Although the rate of mandibular second molar protraction was slightly higher in the late piezocision group, compared to the early one, the duration of space closure in both piezocision groups was similar at the end of treatment, and was faster in both piezocision groups, compared to no piezocision group. This finding was in agreement with previous studies^8^ ^,^ ^11^ ^,^ ^12^ ^-^ ^14^ that reported reduction in treatment time up to 59% by piezocision, and in disagreement with others that reported no difference in treatment time.^9^ ^,^ ^10^

Even though the rate of molar protraction was increased following early and late piezocision groups, the net reduction in space closure duration was less than one month. This was in agreement with Tunçer et al.,^10^ who reported that treatment duration was comparable to the control group, although the piezocision group demonstrated a higher average amount of space closure. Also, the result of the current study confirmed previously reported results by Al-Areiqi et al.,^15^ who suggested that reduction of overall treatment duration in piezocision group was around one month only. On the other hand, this finding contradicts previous reports of up to 43% reduction in treatment duration.^8^ ^,^ ^12^ ^,^ ^14^

In the current study, anchorage loss was comparable between piezocision and no piezocision groups. The result of this study is in agreement with previously published research^6,11^ that reported similar anchorage loss between piezocision and control sides during canine retrac tion. On the other hand, Aksakalli et al. ^7^ reported greater anchorage loss on the control side, compared to the piezocision side. However, none of the reported studies investigated mandibular molar protraction. The finding of the current study might be explained by the action of opposing forces on mandibular second premolars: distal premolar moving forces from the piezocision cut and mesial moving forces acting on the main archwire from the NiTi coil spring attached to the miniscrew. These two opposite forces may have masked anchorage loss. 

Although temporary anchorage devices (TADs) have been proved to be effective in providing absolute anchorage during second molar protraction,^27^ it has been reported that they do not remain absolutely stationary throughout orthodontic loading.^28^ Liou et al.^28^ suggested that miniscrews tips forward by 0.4mm at the screw head site. In the current study, miniscrews were used for superimposition as it was the most stable structure available clinically in the mandibular arch. 

## LIMITATIONS

Limitations of the current study included:

- » A greater female to male ratio.
- » Molar protraction was carried out using insufficient length power arms, to reduce molar tipping.
- » Superimposition was carried out using the miniscrew as a reference point, although it is not absolutely stationary.

## CONCLUSIONS

Early and late piezocision produced similar acceleratory effect and increased second molar protraction temporarily in the first 2-3 months after surgery. Duration of mandibular first molar space closure was reduced by one month only when piezocision was applied. Anchorage loss was similar in the three investigated groups.

## GENERALIZABILITY

Although piezocision increased mandibular second molar protraction, treatment duration was reduced by one month only. Similar effects are produced whether piezocision is performed before molar protraction or three months later. 
